# Rejuvenating the failing heart: multidimensional insights into young blood-mediated anti-aging pathways

**DOI:** 10.3389/fendo.2025.1653567

**Published:** 2025-09-03

**Authors:** Ming-Tai Chen, Rao-Qiong Wang, Yu-Mei Qian, Ting Peng, Xiao-Yu Lan, Ling-Ling Liang, Gang Luo, Qiu-Yu Liu, Meng-Nan Liu

**Affiliations:** ^1^ Department of Cardiovascular Disease, Shenzhen Traditional Chinese Medicine Hospital, Shenzhen, China; ^2^ The Affiliated Traditional Chinese Medicine Hospital, Southwest Medical University, Luzhou, China; ^3^ School of Pharmacy, Southwest Medical University, Luzhou, China

**Keywords:** heart failure, young blood, circulating factors, cardiac repair, anti-aging pathways

## Abstract

Heart failure (HF), a serious stage of many cardiovascular illnesses associated with high morbidity and mortality, has emerged a major global public health concern worldwide. Its key pathophysiological mechanisms include cardiac remodeling, neurohormonal dysregulation and cardiac dysfunction. Despite established clinical treatments, adverse reactions and limited efficacy remain problems. In recent years, great emphasis has been paid to young blood, which is defined as blood from young people or in a youth associated physiological condition, and is abundant in various components that help maintain a youthful state of the organism. Currently, some progress has been made in the study of the anti-aging effects and mechanisms of young blood in older individuals, indicating therapeutic potential in the treatment of age-related diseases. In this paper, the major pathophysiological mechanisms of HF, the rejuvenating circulating factors in young blood and their positive effects on aging tissues are summarized. Moreover, the rejuvenating effects of young blood on the failing heart and the possible mechanisms of its action from multiple perspectives are investigated and discussed, aiming to provide theoretical foundation and potential therapeutic targets for the treatment of HF related diseases.

## 1 Introduction

Heart failure (HF) is a life-threatening clinical syndrome and a critical manifestation of multiple cardiovascular diseases. Marked by high incidence and mortality, it has become a major global public health concern. Epidemiological data reveal that the global prevalence of HF ranges from 1% to 10%, with an estimated incidence of 1 to 20 cases per 1000 person-years (1). Recognized risk factors for HF include hypertension, diabetes, coronary artery disease, obesity and smoking (2). Clinically, HF is primarily treated with diuretics, β-blockers and angiotensin-converting enzyme inhibitors, which have proven clinical efficacy (3). However, due to the high incidence of adverse reactions and recurrence rates associated with drug therapy, as well as the fact that some patients still fail to achieve ideal treatment outcomes after other standardized treatments, efforts are being actively made to explore and develop new drugs and alternative therapeutic approaches beyond pharmacotherapy.

Young blood refers to blood from young individuals or those exhibiting a physiologically youthful condition, whole blood including cells and plasma (cell-free, contains clotting factors), characterized by cellular and molecular compositions that facilitate the preservation of a youthful homeostasis. Recent studies employing heterochronic parabiosis-a technique that combines the circulatory systems of animals of varying ages to investigate systemic effects on aging and age-related diseases (4–6). They have shown that blood from young mice can enhance the functional capacity of aging tissues and organs, such as the liver, heart, brain and muscles (7, 8). The geroprotective qualities and specific mechanisms of young blood in aged organisms are progressively being clarified, demonstrating significant therapeutic potential for age-related illnesses (9–12). Given that the mechanisms underlying the association between young blood and HF have not yet been systematically integrated, this review aims to integrate the pathophysiological mechanisms of HF with the reparative effects and potential molecular mechanisms of young blood on the failing heart, so as to provide a systematic reference for clinical translation in this field.

## 2 Pathophysiological mechanisms of HF

**Figure 1:**
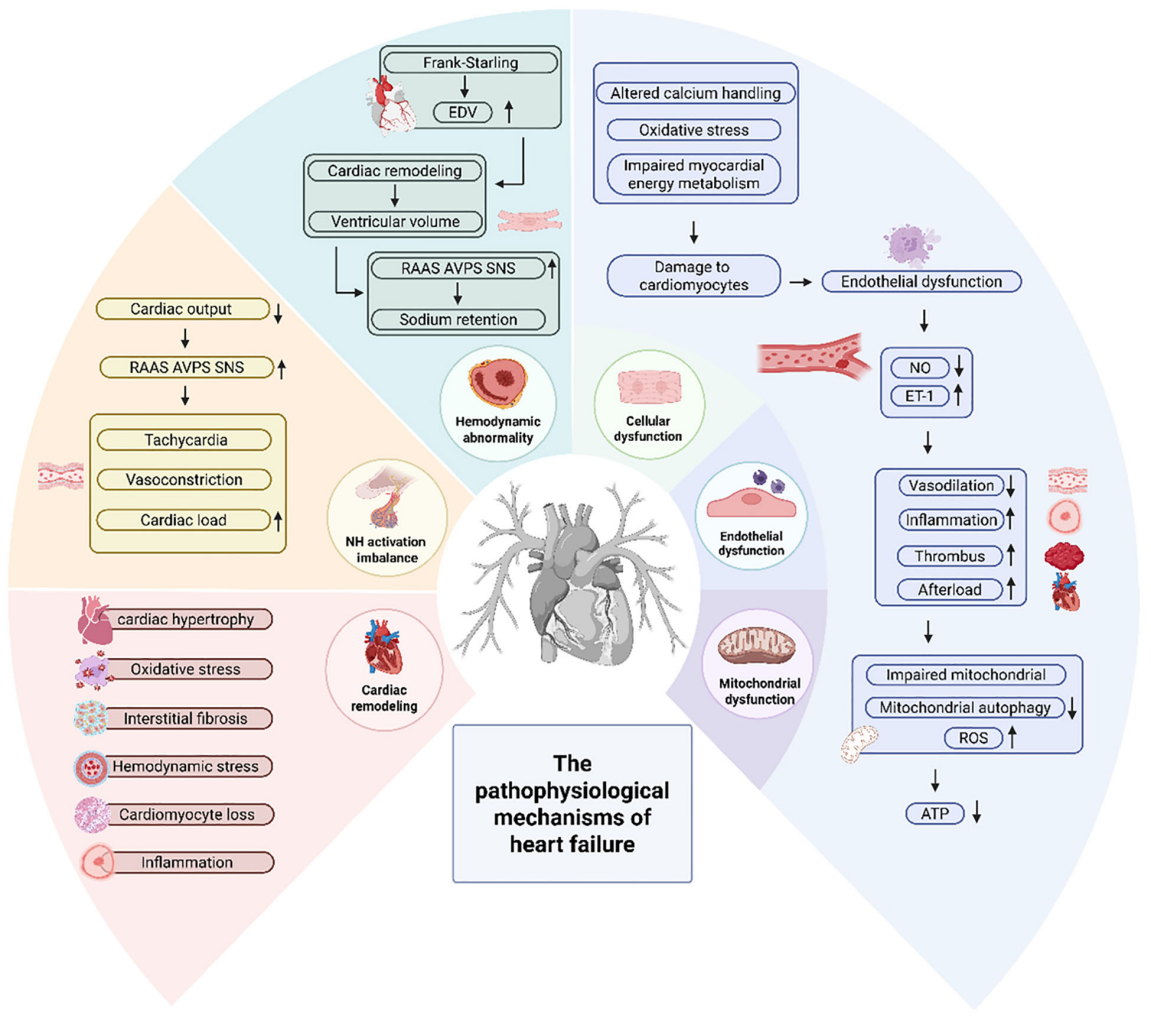
The pathophysiological mechanisms of HF.

HF is a clinical syndrome with diverse etiologies (13), characterized by diminished effective cardiac output (14), and manifested as symptoms such as dyspnea, fluid retention, nausea, vomiting and syncope (15). The primary causes are coronary artery disease, hypertension, valvular heart disease and other factors (16–18). The pathophysiological mechanisms of HF are illustrated in **Figure 1**.

### 2.1 Cardiac remodeling

Cardiac remodeling is frequently regarded as a pivotal factor influencing the clinical progression of HF. Cardiac remodeling is defined as changes in genomic expression leading to molecular, cellular and interstitial modifications, clinically evident as an increase in cardiac volume due to hemodynamic overload or injury, and a transformation in cardiac shape from elliptical to spherical, resulting in alterations in ventricular dimensions and both systolic and diastolic dysfunction (14, 19, 20). Cardiac remodeling is influenced by hemodynamic stress, neurohormonal activation and additional mechanisms now under investigation. The key steps leading to cardiac remodeling include cardiac hypertrophy, cardiomyocyte loss and interstitial fibrosis. During this remodeling, fibroblasts differentiate into myofibroblasts, activating their secretory functions and producing large amounts of extracellular matrix proteins. This results in scar tissue formation, which impedes the normal contractile function of cardiomyocytes, ultimately contributing to the development of HF in the long term (21, 22). Additional components involved include the interstitium, collagen and the coronary vascular system, with associated processes including ischaemia, cell necrosis and apoptosis. Patients with severe remodeling show progressive deterioration of cardiac function, with severe cases progressing to HF stages. Nonetheless, factors beyond remodeling can also influence the course of heart disease, and disease progression may occur in other ways in the absence of cardiac remodeling (19).

### 2.2 Neurohormonal activation imbalance

As a result of reduced cardiac output several neuroendocrine systems are activated, including the renin-angiotensin-aldosterone system (RAAS), arginine vasopressin system (AVPS) and the sympathetic nervous system (SNS). The persistent neurohormonal activation of the RAAS, AVPS and SNS plays a key role in the progression of HF. Extended activation of these systems leads to tachycardia, increased systemic vascular resistance, vasoconstriction, sodium and water retention, which in turn increases cardiac load and exacerbates HF. In addition, it leads to polyadrenergic receptor desensitization, cardiomyocyte hypertrophy, necrosis, cellular dysmorphism, myocardial fibrosis, renal arteriolar and venous vasoconstriction, diminished response to natriuretic peptides (NPs), peripheral vasoconstriction and vascular hypertrophy, and cardiac arrhythmia et al. (20, 23).

### 2.3 Hemodynamic abnormality

HF is characterized by pathological hemodynamic disturbances, including elevated cardiac filling pressures, which is associated with reduced cardiac output. The hemodynamic abnormalities in HF are not only influenced by cardiomyocytes, but also include important processes such as pericardial restriction, ventricular interactions and altered venous volumes (24). As the failing heart attempts to maintain adequate function, several compensatory mechanisms occur to ensure relatively normal cardiac output and maintain adequate cardiac function, including increased cardiac output and ventricular end-diastolic volume via the Frank-Starling mechanism, which increases cardiac output and cardiac workload, escalated ventricular volume and wall thickness via ventricular remodeling and maintained tissue perfusion by activating the neurohormonal system to increase mean arterial pressure, leading to increased myocardial contractility and sodium and water retention (25). The initial augmentation of ventricular wall thickness compensates for the heightened workload. However, it progressively diminishes cardiac diastolic function and leads to HF (16). These compensatory mechanisms, while initially beneficial in the early stages of HF, eventually lead to a vicious cycle of worsening HF (25). In addition, cardiac remodeling-induced cardiac changes can impair systolic function and reduce stroke volume output (26). Additionally, myocardial fibrosis, valve dysfunction and arrhythmias can also compromise systolic and diastolic function, thereby affecting cardiac output (16).

### 2.4 Cellular dysfunction

Decreased and impaired function of cardiomyocytes, including altered calcium handling and energy production (14), leads to impaired cardiac contractile function, cardiomyocyte apoptosis, myocardial ischemia and myocardial fibrosis. Among them, myocardial fibrosis destroys myocardial structure and leads to myocardial disorders, affecting the systolic and diastolic functions of the heart, leading to structural changes and inducing arrhythmias (27). Moreover, myocardial infarction significantly contributes to ventricular remodeling and the subsequent development of HF (28). Damage to cardiomyocytes involving inflammation, oxidative stress and impaired myocardial energy metabolism, they also are important causes of HF (14). Inflammation contributes to the pathogenesis and progression of HF through different mechanistic pathways. The HF state is characterized by an imbalance between pro-inflammatory and anti-inflammatory cytokines (29). The pro-inflammatory cytokines interleukin-1 (IL-1) and tumor necrosis factor-α (TNF-α) both induce systolic and diastolic dysfunction, and the latter also promotes adverse cardiac remodeling (30). Oxidative stress characterized by the generation of reactive oxygen species (ROS) and intrinsic energy metabolism. The generation of ROS and endogenous antioxidant defense systems are significant factors in the pathogenesis of cardiac remodeling and HF (31). When existing at low concentrations, ROS exert a pivotal function in maintaining cellular homeostasis. Nevertheless, an excess of ROS can lead to cellular dysregulation, lipid peroxidation, cellular injury and apoptosis. Ultimately, through the induction of cardiac fibroblast proliferation and the activation of matrix metalloproteinases, it triggers extracellular matrix remodeling, displaying profibrogenic properties. This process significantly influences the onset and progression of HF, contributing to the exacerbation of the pathological state (32). Van Bilsen et al. proposed the concept of myocardial metabolic remodeling in the context of myocardial energy metabolism disorders. They posited that HF induces a disturbance in the metabolism of carbohydrates and fats within myocardial cells, leading to alterations in the cardiac energy metabolic pathway and resulting in the abnormal structure and function of myocardial cells (33). There is increasing evidence that impaired myocardial energy metabolism can be one of the causes of HF, and HF can also exacerbate impaired myocardial energy metabolism. Therefore, reducing excessive myocardial energy expenditure and improving myocardial energy metabolism can improve the prognosis of HF (34).

### 2.5 Endothelial dysfunction

Endothelial cells are a monolayer of cells that cover the inner surface of blood vessels and act as a functional and structural barrier between the blood and the vessel wall, preventing platelets and leukocytes from adhering and aggregating, controlling permeability to plasma constituents, and regulating blood flow (35). Endothelial dysfunction, characterized by an imbalance in nitric oxide (NO, be defined in subsequent use) production and an increase in endothelin-1, leads to a shift in endothelial function toward reduced vasodilation, a pro-inflammatory state and pro-thrombotic properties. This is also one of the early mechanisms determining the decrease in organ perfusion, intolerance to physical exertion and the progression of HF. Additionally, it is associated with cardiovascular diseases such as coronary artery disease, hypertension and chronic HF (36–38). It leads to impaired vasodilation and compromised coronary blood flow and increased vascular resistance. This endothelial dysfunction leads to increased afterload, which puts additional stress on the heart. Some studies have shown that inflammation also induces endothelial dysfunction (16).

### 2.6 Mitochondrial dysfunction

Mitochondria are key double-membrane organelles for aerobic respiration in eukaryotic cells, mainly producing adenosine triphosphate (ATP) to provide energy for the organism, and participating in processes such as calcium homeostasis, lipid synthesis and regulation of apoptosis (39, 40). The heart, as the most metabolically active organ with the highest mitochondrial content, relies mainly on ATP generated by fatty acid oxidative metabolism to provide energy to maintain cardiac systolic and diastolic functions under physiological conditions. Bioenergetic homeostasis is accomplished almost exclusively through the “energy web” composed of the mitochondrial network and its associated phosphate transfer pairs (39). Common mitochondrial dysfunctions include decreased bioenergetics, increased oxidative stress, dysregulation of calcium homeostasis and altered mitochondrial dynamics (41). Mitochondrial dysfunction is thought to be one of the important pathogenetic mechanisms affecting cardiovascular diseases and is mainly associated with mitochondrial DNA (mtDNA) mutations, impaired mitochondrial autophagy, and elevated levels of mitochondria-derived ROS. Specifically, mitochondrial dysfunction has an important role in atherosclerosis, ischemic or reperfusion injury, calcium dyshomeostasis, neurodegenerative diseases, and cardiac systolic-diastolic dysfunction (41, 42).

## 3 Pharmacological treatment of HF

Clinical treatment options vary significantly depending on the type of HF patient, comorbidities and other clinical conditions (43). According to the 2022 Heart Failure Management Guidelines jointly issued by the American College of Cardiology/American Heart Association/American Heart Failure Society (AHA/ACC/HFSA), HF is classified based on left ventricular ejection fraction (LVEF), for HF with reduced ejection fraction (HFrEF, LVEF<40%, characterized by ventricular systolic dysfunction and progressive cardiac remodeling), renin-angiotensin system inhibitors (RASI), including angiotensin receptor–neprilysin inhibitor (ARNI), angiotensin-converting enzyme inhibitor (ACEI), angiotensin receptor blockers (ARB), mineralocorticoid receptor antagonist (MRA), sodium-dependent glucose transporters 2 inhibitors (SGLT2i) and β-receptor blocker. For HF with mildly reduced ejection fraction (HFmrEF, 40%≤LVEF ≤ 49%, possesses some characteristics of both HFrEF and HFpEF), SGLT2i is recommended in class 2a, ARNI, ACEI, ARB, MRA, and β-receptor blocker are recommended in class 2b. For HF with preserved ejection fraction (HFpEF, LVEF≥50%, mainly manifested by diastolic dysfunction, endothelial dysfunction, etc.), SGLT2i is recommended in class 2a, MRA and ARNI are recommended in class 2b. For HF with improved ejection fraction (HFiEF, previously HFrEF, with LVEF improved to ≥40% after treatment), ARNI, ACEI, ARB, MRA and β-receptor blockers (44). While guideline-directed pharmacological treatment can improve patient survival and quality of life, the burden of mortality in HF remains high. Pharmacological treatments are mainly symptom-directed, may lead to suboptimal stratification of treatment strategies, limited therapeutic efficacy, increased adverse events, and the emergence of drug resistance. These challenges collectively place patients at risk of progressive disease deterioration and poor clinical outcomes, so there is a need to look for more ideas and approaches to treating HF in order to achieve better outcomes (3, 45), and young blood offers a promising new avenue.

## 4 Therapeutic targets of young blood

Young blood contains a spectrum of circulating factors with rejuvenating properties, which play critical roles in sustaining physiological homeostasis, alleviating HF and other aging-related diseases, and promoting tissue and organ functional recovery. The therapeutic targets of young blood are shown in **Table 1**.

**Table 1:** The therapeutic targets of young blood.

Key factor	Model	Intervention method	Regulatory mechanism	Function	References
PEDF	*In vivo*, aging induced functional decline and pathology mice.	Subcutaneously inject recombinant PEDF 4 µg every day for 6 weeks.	TIMP2 ↑, Igf2 ↑, Ptgds ↑, Foxm1 ↑, Prkaa1 ↑, Glul ↑, Trem2 ↑, Fcer1g ↑, Siglec1 ↑	Improve cognition in aged mice, reduce liver fibrosis, counter age-related renal lipotoxicity.	(46)
	*In vivo*, human umbilical vein endothelial cells exposed to TNF-α model.	Incubate 10 ng/mL TNF-α for 24 h.	ROS ↓, NF-κB ↓, IL-6 ↓	Reduce inflammation and oxidative stress, resist atherosclerosis formation.	(47)
EV	*In vivo*, aging induced functional decline and diseases mice.	Intravenously inject young sEVs 200μL seven times for 2 weeks.	PGC-1α ↑, ATP ↑, p21 ↓, p16 ↓	Alleviate aging at the molecular, mitochondrial, cellular, and physiological levels.	(48)
	*In vivo*, FeCl3 induced carotid artery thrombosis mice.	Inject 50 μg purified platelet-derived exosomes 10 min prior to induction of thrombosis.	CD36 ↓, NO_2_-LDL ↓	Inhibit platelet activation and atherosclerotic thrombus formation.	(49)
Klotho	*In vitro*, LPS induced acute lung injury in A549 cell.	Inject recombinant human klotho protein 400 pM for 1 h.	NLRP3 ↓, TNF-α ↓, IL-1β ↓, IL-6 ↓,SOD ↑, GSH-Px ↑, Bcl-2 ↑, Bax ↓, SIRT1↑, Nrf2 ↑	Alleviate LPS induced inflammatory injury and oxidative stress of A549 cells, restore mitochondrial function.	(50)
	*In vivo*, EMT induced subretinal fibrosis mice.	Inject recombinant human klotho protein 1 μL 10 or 20 nM for 4 days.	EMT ↓, ZO-1 ↑, α-SMA ↓, N-cad ↓, c-Myc, ↓ cyclinD1 ↓, β-catenin ↓, ERK1 ↓, ERK2 ↓	Against subretinal fibrosis.	(51)
GDF11	*In vivo*, aging induced depression-like phenotype mice.	Inject rGDF11 1 mg/kg every night for 3 weeks.	Sox2 ↑, SA-βGal ↓, FoxO3a ↑, mTOR ↓, Beclin 1 ↑	Enhance memory and cognitive ability, alleviate symptoms of aging and depression.	(52)
	*In vivo*, T1DM and T2DM induced wounds in mice.	Inject rGDF11 50 ng/mL,10 μL twice a day for 14 days.	HIF-1ɑ ↑, VEGF ↑, SDF-1ɑ ↑	Stimulate the mobilization of endothelial progenitor cells to the injured area, promote neovascularization and wound healing.	(53)
TIMP2	*In vivo*, aging induced decline in nervous system function mice.	Intravenously inject human umbilical cord plasma 175 μL for 2 weeks.	c-Fos ↑, Egr1 ↑, Junb ↑, Camk2a ↑, Bdnf ↑, Ntf3 ↑, Ppp3ca ↑, Nptx2 ↑	Revitalize hippocampal function in aged mice.	(54)
	*In vivo*, myocardial infarction induced cardiac remodeling mice.	Knock out TIMP2.	MT1-MMP ↑, MCP-1 ↑, IL-6 ↑, MMP2 ↓	Accelerate the development of cardiac remodeling after myocardial infarction in mice.	(55)
	*In vivo*, col1α1 fibroblasts induced intracerebral hemorrhage mice.	Infuse recombinant human TIMP2 0.8 μg over 4 days.	ZO-1 ↑, claudin5 ↑, occludin ↑	Repair blood-brain barrier damage and hemorrhagic brain injury.	(56)
NO	*In vitro*, eNOS knocked down in human umbilical vein endothelial cells.	Knock down eNOS.	p21 ↓, Ki67 ↑, AKT ↓, ERK ↑	Induce cell migration and proliferation, inhibit formation of tube-like structures *in vitro*.	(57)
	*In vivo*, healthy adults.	Intravenously infuse SMTC 3.0 µmol/kg over 10 min.	nNOS ↓	Regulate the cerebral blood flow and regional cerebral perfusion in the human hippocampal region.	(58)
HDL	*In vivo*, mechanical stress induced cardiomyocyte autophagy and cardiac hypertrophy mice.	Pre-administer HDL 100 μg/mL for 30 min.	AT1 receptor ↓, ANP ↓, SAA ↓, LC3b-II ↓, beclin-1 ↓	Inhibit mechanical stress-induced cardiomyocyte autophagy and cardiac hypertrophy	(59)
	*In vivo*, oxygen and glucose deprivation induced cardiomyocytes necrosis mice.	Incubate with 100 µg /mL human HDL for 30 min.	AKT1 ↑, AKT2 ↑	Protect cardiomyocytes against necrosis.	(60)
	*In vivo*, ischemia reperfusion injury induced necrosis mice.	Perfuse rHDL 140μg/mL for 7min.	ERK1 ↑, ERK2 ↑, STAT3 ↑, AKT ↑	Protect heart and reduce ischemia-reperfusion injury.	(61)
PF4	*In vivo*, inflammation induced aging disease mice.	Intravenously inject PF4 5 μg/mL over 24 days.	Nfkb1 ↓, Il1b ↓, CD11b ↓, Bdnf ↑, Ntf3 ↑, CCL2 ↓, TNF ↓, CyPA ↓	Alleviate neuroinflammation, induce synaptic plasticity-related enhancement, rejuvenate the ageing immune system.	(62)
	*In vivo*, aging induced cognitive deficits in the brain mice.	Subcutaneously inject klotho 10 µg /kg for 4 h.	PF4 ↑, Akap11 ↓, Arhgef9 ↓, Mecp2 ↓	Enhance cognition in the young brain and decrease cognitive deficits in the aging brain.	(63)
HSA	*In vitro*, HF patients.	Infuse serum albumin≤ 2.9 g/dL for 24 h.	HAS ↑	Increase risk-adjusted mortality during hospitalization, prolong stays in hospital.	(64)

### 4.1 Systemic mediators of young blood action

#### 4.1.1 Pigment epithelium-derived factor

Among the serine protease inhibitor superfamily, PEDF has attracted attention as a 50-kDa secreted glycoprotein that exerts different physiological activities in various tissues, especially reducing myocardial fibrosis in HF while promoting cardiomyocyte apoptosis, thereby accelerating the progression of HF (65–67). Studies in animal models of injury and disease have shown that PEDF is involved in the maintenance of physiological homeostasis through its proven neurotrophic, antiangiogenic, immunomodulatory, tumor-killing and stem cell support functions. Meanwhile, PEDF induces a protective transcriptional profile in the hippocampus, liver and kidney of aged mice, improving cognitive function as well as liver and kidney integrity, suggesting that maintaining or supplementing PEDF levels in the elderly may have therapeutic implications for age-related diseases and chronic disorders of these tissues (46). Sho-Ichi Yamagishi et al. demonstrated that PEDF exerts anti-inflammatory, antioxidant and anti-atherosclerotic effects by inhibiting TNF-α-induced IL-6 expression through inhibition of nicotinamide adenine dinucleotide phosphate (NADPH) oxidase-mediated ROS generation (47).

#### 4.1.2 Extracellular vesicles

EVs are a heterogeneous group of nanoscale membrane vesicles composed of protein-rich lipid bilayers and closed proteins and RNAs originating from EV-producing cells that circulate throughout the body via the bloodstream and act as messengers in intercellular communication networks (48). Importantly, EVs plays critical therapeutic roles in HF through pro-angiogenic and cardiac remodeling-alleviating (68). EV-mediated communication is integral to the homeostasis of tissues and organs and significantly influences pathological processes (69). Xiaorui Chen et al. showed that young EVs have pleiotropic effects on systemic physiology and homeostasis, such as improving the morphology and function of aging tissues and organs, enhancing endurance and cognitive ability in aged mice, and improving mitochondrial dysfunction in aged cells, which may therefore explain the rejuvenating effects of young blood (48). Raghu Kalluri et al. observed that EVs can be classified into two main categories, extracellular vesicles and exosomes, among which exosomes are associated with immune responses, metabolic diseases and neurodegenerative diseases (70). Sowmya Srikanthan et al. demonstrated that platelet-derived exosomes inhibit atherosclerotic thrombosis by reducing CD36-dependent lipid loading in macrophages and suppressing platelet activation (49).

### 4.2 Key factors in young blood

#### 4.2.1 Klotho

Klotho, an anti-aging gene, is expressed predominantly in the epithelial cells of the distal renal tubules of the kidney and in the choroid plexus of the brain (71). Yuechi Xu et al. showed that the human klotho gene encodes α-klotho, a multifunctional protein that includes three types: full-length transmembrane α-Klotho, truncated soluble α-Klotho, and secreted α-Klotho (72). Studies have shown that Klotho exerts a positive effect in HF by reducing inflammation, improving cardiac function and alleviating mitochondrial dysfunction (73, 74). Yanjun Zeng et al. demonstrated that Klotho inhibits lipopolysaccharide (LPS)-induced NOD-like receptor family pyrin domain containing 3 (NLRP3) inflammatory vesicle activation in A549 cells via the activation of the Sirtuin 1 (SIRT1)/nuclear factor erythroid 2-related factor 2 (Nrf2) signaling pathway. This process mitigates LPS-induced inflammatory injury in A549 cells and restores mitochondrial function (50). Yingle Jiang et al. showed that Klotho inhibits extracellular signal-regulated kinase (ERK)1/2 and wingless/integrated (Wnt)/β-catenin signaling pathway and attenuates the epithelial-mesenchymal transition of retinal pigment epithelial cells in subretinal fibrosis, thus exerting an anti-inflammatory effect on subretinal fibrosis (51).

#### 4.2.2 Growth differentiation factor-11

GDF11 belongs to the transforming growth factor β superfamily, which plays a key role in embryonic development and is a critical regulator of a wide range of tissue patterning and formation (52). Carine Moigneu et al. demonstrated that GDF11 can act directly on hippocampal neurons *in vitro* to improve memory and alleviate aging and depression-like symptoms (52), while GDF11 deficient in cardiomyocytes leads to cardiac remodeling and eventually HF under pressure overload (75). Ying Zhang et al. showed that GDF11 promotes neovascularization and enhances diabetic wound healing by stimulating the mobilization of endothelial progenitor cells to injured areas mediated by the hypoxia-inducible factor (HIF)-1ɑ-vascular endothelial growth factor (VEGF)/stromal cell-derived factor (SDF)-1ɑ pathway (53). In addition, GDF11 has the ability to regulate muscle aging, reverse age-related decline in skeletal muscle function, improve mature adipocyte metabolism and anti-inflammatory properties (76–78).

#### 4.2.3 TIMP2

TIMP2 holds a significant role within the family of tissue inhibitors of metalloproteinases, is highly expressed in the myocardium, and its deficiency results in exacerbate ventricular remodeling leading to severe HF (79). CASTELLANO et al. showed that human umbilical cord plasma and young rat plasma contain high levels of the anti-aging protein tissue TIMP2. Their study found that injection of human umbilical cord plasma into the hippocampus of aged mice activated this brain region, enhanced the cognitive function, promoted neuronal synapses formation, and improved the neuronal function in the hippocampus of the aged mice (54). Vijay Kandalam et al. demonstrate that TIMP2 defect promotes cardiac remodeling following myocardial infarction in mice by enhancing the activity of membrane type 1 matrix metalloproteinase (MT1-MMP) (6, 55). Lingling Xu et al. demonstrated that TIMP2 partially mediates fibroblasts to repair blood-brain barrier damage and hemorrhagic injury (56).

#### 4.2.4 NO

NO is a highly reactive and diffusible, gaseous free radical, synthesized by three different isoforms of NO synthase (NOS): neuronal NOS (nNOS), inducible NOS (iNOS) and endothelial NOS (eNOS) (80, 81). The neurotransmitter function of NO depends on dynamic regulation of NOS (82). Shuhan Bu et al. demonstrated that knockdown of eNOS induced endothelial cell metastasis and proliferation through activation of mitogen-activated protein kinase (MAPK)/extracellular signal-regulated kinase (ERK) and inhibition of the phosphatidylinositol 3-kinase (PI3K)/AKT (protein kinase B, the key downstream target protein of PI3K) pathway, and inhibited the formation of tubular structures *in vitro*, confirming that eNOS regulates endothelial function by inversely controlling the proliferation and migration of endothelial cells as well as by directly regulating their tube-forming potential (57). Furthermore, Kevin O’Gallagher et al. demonstrated that nNOS modulates cerebral blood flow and local cerebral perfusion in the human hippocampal region through the administration of the nNOS inhibitor, S-methyl-L-thiotreponine (SMTC), which resulted in decreased cerebral blood flow (58). In addition, NO influences the progression of HF through alleviating vascular endothelial dysfunction, and influencing the nervous system and hemodynamics (83, 84).

#### 4.2.5 High-density lipoprotein

HDL is a complex lipoprotein structure composed of lipids and proteins, along with the regulatory factors they transport, its level is related to the risk of HF (85). Lin Li et al. showed that HDL inhibits mechanical stress-induced cardiomyocyte autophagy and cardiac hypertrophy through the angiotensin II type 1 receptor-mediated PI3K/AKT pathway (59). Kristina K Durham et al. demonstrated that HDL protects cardiomyocytes from oxygen and glucose deprivation-induced necrosis via scavenger receptor class B, type 1 (SR-B1), PI3K, and AKT1 and AKT2 pathways (60). In addition, Marie-Claude Brulhart-Meynet et al. displayed that remodeled HDL mediated by sphingosine-1-phosphate (S1P) exerts a cardioprotective effect and mitigates ischemia-reperfusion injury through activation of ERK1/2, signal transducer and activator of transcription 3 (STAT3) and AKT (61).

#### 4.2.6 Platelet factor 4

PF4 is a chemokine secreted by platelets, playing a role in coagulation and immunomodulation (86). PF4 indirectly influences the development of HF by impairing endothelial function and promoting atherosclerotic lesions (62). A study by Adam B Schroer et al. detected higher levels of PF4 in platelet fractions from young mice than from old mice by western blot analysis. They showed that PF4 has the ability to attenuate age-related neuroinflammation, induce synaptic plasticity-related enhancement, rejuvenate the aging immune system and rescue cognition in old age (86). Cana Park et al. showed that klotho activates platelet release of PF4 and restores the expression of aging and cognition-related factors in the hippocampus, thereby enhancing cognitive performance in young brains and reducing cognitive deficits in older brains (63).

#### 4.2.7 Human serum albumin

HSA is the predominant serum (plasma without fibrinogen) protein and has been shown to have antioxidant properties (6, 87). Yali Wang et al. found that serum albumin levels are negatively correlated with the risk of atrial fibrillation, indicating that HSA may reduce the risk of atrial fibrillation (88). Hypoalbuminemia is prevalent in patients with HF and reduced albumin levels are associated with an increased prevalence of HFpEF, however, Tongqing Yao et al. found through data analysis that albumin therapy for HF carries certain risks (be showed in **Table 1**) (64). Lucia M Ruiz-Perera et al. experimentally found that human plasma and its component HSA play an important protective role against neuronal death induced by oxidative stress (6, 89).

## 5 Multidimensional analysis of cardiac repair mediated by young blood

**Figure 2:**
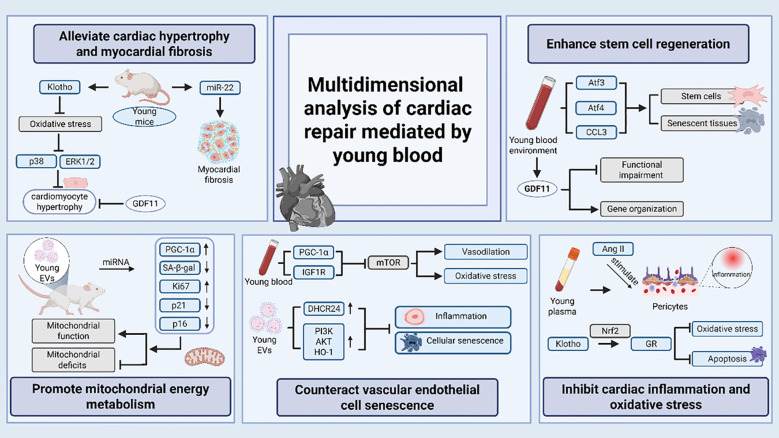
The specific mechanism of action of young blood.

The multiple circulating factors in young blood do not act in isolation, they exert positive influence on cardiac health through various mechanisms and play an important role in regulating cardiac physiological functions and improving pathological conditions. The multidimensional analysis of cardiac repair mediated by young blood is shown in **Figure 2** and **Table 2**.

**Table 2:** The multidimensional analysis of cardiac repair mediated by young blood.

Model	Intervention method	Primary control mechanism	Function	References
*In vivo*, aging induced cardiac hypertrophy mice.	Intraperitoneally inject rGDF11 0.1 mg/kg every day for 30 days.	GDF11 ↑, TGFβ ↑, pSMAD2 ↑, pSMAD3 ↑, BNP ↓, ANP ↓	Reduce the degree of cardiac hypertrophy.	(90)
*In vivo*, indoxyl sulfate induced myocardial cell hypertrophy mice.	Inject Klotho 400 pmol/L for pretreatment 1 h.	Klotho ↑, Nox2 ↓, Nox4 ↓, ROS ↓, TRPC6 ↓	Inhibit myocardial cell hypertrophy induced by indoxyl sulfate.	(91)
*In vivo*, ischemia induced myocardial cell apoptosis mice.	Inject 1 µg exosomes after LAD.	miR-22 ↑, Mecp2 ↓	Reduce the apoptosis of ischemic myocardial cells, alleviate fibrosis and enhance the cardiac function.	(92)
*In vivo*, Klotho deficiency induced heart aging mice.	Intraperitoneally inject Klotho 100 mg/kg every day for 2 weeks.	Klotho ↑, Nrf2 ↑, GR ↑, ANP ↓, α-MHC ↓, ROS ↓	Alleviate cardiac fibrosis, improve diastolic dysfunction in the aging heart and inhibit oxidative stress.	(93)
*In vivo*, aging induced the functional impairment of muscle stem cells mice.	Intraperitoneally inject rGDF11 0.1 mg/kg every day for 4 weeks.	GDF11 ↑, PGC-1α ↑	Improve the functional impairment of aged muscle stem cells and restore the integrity of the genetic tissue.	(94)
*In vivo*, aging induced mitochondrial deficits mice.	Intravenously inject young sEVs 200μL seven times for 2 weeks.	young EVs ↑, PGC1-α ↑, SA-β-gal ↓, Ki67 ↑, p21 ↓, p16 ↓	Improve mitochondrial function and alleviate mitochondrial deficits in aged tissues.	(48)
*In vivo*, NO-induced endothelium-dependent vasorelaxation mice.	Subcutaneously inject Banamine 2 mg/kg twice a day for 3 days and then once daily for 4 days.	PGC-1α ↑, IGF1R ↑, mTOR ↓	Rescue NO-mediated endothelium-dependent vasodilation and attenuate vascular oxidative stress.	(95)
*In vivo*, TNF-α induced inflammation in HCAECs rabbit.	Intravenously infuse lipid-free apoA-I 8 mg/kg 24 h in advance.	rHDL ↑, DHCR24 ↑, HO-1 ↑	Inhibit vascular endothelial inflammation.	(96)
*In vivo*, ischemia - reperfusion induced acute kidney injury mice.	Inject plasma retroorbitally 100 uL once every two days within 28 days.	Ccl19 ↓, Ccl21 ↓, Cxcl13 ↓	Ameliorate the pro-inflammatory and pro-fibrotic effect of angiotensin II on pericytes.	(97)
*In vivo*, myocardial I/R injury induced cell death mice.	Intravenous bolus of exosomes 0.4 μg/mL 5 min in advance.	ATP ↑, NADH ↑	Restore energy consumption and reduce oxidative stress and inflammation.	(98)

### 5.1 Alleviate cardiac hypertrophy and myocardial fibrosis

Cardiac hypertrophy is the result of compensatory hypertrophy of myocardial tissue, which is divided into physiological hypertrophy and pathological hypertrophy. In physiological hypertrophy, the heart has normal or enhanced contractile function, reversible hypertrophy, and intact energy metabolism without causing HF. In pathological hypertrophy, the heart is associated with cardiomyocyte death, myocardial fibrosis, incomplete reversibility of hypertrophy, and metabolic remodeling of the myocardium, which ultimately leads to systolic dysfunction and HF (99). Francesco S Loffredo et al. demonstrated that heterochronic parabiosis is able to reverse age-related cardiac hypertrophy. Further screening studies showed that GDF11 is a mediator of systemic anti-hypertrophic activity in young mice and has powerful anti-hypertrophic properties (90). Ke Yang et al. found that Klotho is predominantly synthesized in the kidneys and subsequently released into the serum. It has antioxidant activity and can inhibit the cardiomyocyte hypertrophy induced by indoxyl sulfate (an important uremic solute) by blocking oxidative stress and inhibiting the signaling pathways of p38 and ERK1/2 (91). Myocardial fibrosis is a pathological process in which excessive deposition of fibrous connective tissue in myocardial tissue leads to structural and functional abnormalities of myocardial fibers, affecting cardiac diastolic function, leading to arrhythmias and increasing the risk of cardiovascular events. Yuliang Feng et al. demonstrated that exosomes secreted by mesenchymal stem cells after ischemic preconditioning contain miR-22 that targets Mecp2. Through this mechanism, these exosomes can significantly reduce apoptosis of ischemic cardiomyocytes and myocardial fibrosis (92). Kai Chen et al. proved that cardiac-specific overexpression of glutathione reductase (a flavoprotein oxidoreductase, GR) attenuates cardiac fibrosis in aged mice, and Klotho secreted by young mice largely ameliorated Klotho deficiency-induced and aging-associated cardiac diastolic dysfunction (71).

### 5.2 Enhance stem cell regeneration

The decline in tissue regenerative capacity is a hallmark of aging and may be due to age-related changes in tissue-specific stem cells. Conboy et al. established paracrine pairs through heterochronic parabiosis and reveal that stem and progenitor cells retained their proliferative potential even in aging, and that serum from young animals reactivated the regeneration of senescent tissues (93). Shuai Ma et al. have found that hematopoietic stem and progenitor cells are sensitive to the environment of young blood by constructing an allogeneic symbiosis model and mapping the single-cell transcriptome, as well as the fact that young blood can induce changes in hematopoietic and immune systems by restoring the levels of key transcription factors, such as activating transcription factor (Atf)3, Atf4 and C-C motif chemokine ligand (CCL3), which promotes the activation of stem cells and systematic rejuvenation of senescent tissues (100). Manisha Sinha et al. demonstrated that supplementation of the body’s GDF11 levels through heterochronic symbiosis or systemic delivery of recombinant proteins reversed functional impairment and restored the integrity of gene organization in senescent muscle stem cells, suggesting an ameliorative effect of GDF11 on age-related skeletal muscle and stem cell dysfunction (76).

### 5.3 Promote mitochondrial energy metabolism

Declining mitochondrial bioenergetics is a characteristic feature of the aging process. Jenny L Gonzalez-Armenta et al. demonstrated that circulating factors themselves can mediate age-related structural and functional changes in mitochondria by using a heterochronic symbiosis model (101). Francisco Alejandro Lagunas-Rangely noted that heterochronic symbiosis of EV in young mice increased the expression of peroxisome proliferator-activated receptor gamma coactivator 1α (PGC-1α) in older mice through its miRNA carriers. These miRNAs respectively target amyloid β precursor protein, poly [ADP-ribose] polymerase 2, and HIF-1α inhibitor, which enhances mitochondrial function and attenuating mitochondrial defects in aging tissues (94). In addition, Xiaorui Chen et al. showed that young EVs stimulate PGC-1α expression both *in vitro* and *in vivo* via their miRNA cargo, improving mitochondrial function and enhancing mitochondrial energy metabolism to ameliorate degenerative changes and age-related dysfunctions, including cardiac (48).

### 5.4 Counteract vascular endothelial cell senescence

Vascular senescence is a degenerative change that occurs in the body with age and is manifested by thickening of the vascular wall, reduction of elastic fibers and dysfunction of endothelial cells. Endothelial cells are essential for vascular homeostasis, maintenance of blood flow, regulation of vascular tone, pro-inflammatory responses and neovascularization. Senescent endothelial cells display a flattened and enlarged morphology, reduced NO bioavailability, and increased secretion of various pro-inflammatory cytokines. The accumulation and deposition of which leads to vascular dysfunction and vascular senescence, and also promotes inflammation, thrombosis and atherosclerosis (102). Tamas Kiss et al. showed that, through a heterotopic symbiosis model, young blood can inhibit mammalian target of rapamycin (mTOR) signaling through activation of the PGC-1α and insulin-like growth factor 1 receptor (IGF1R) -mediated pathway and inhibiting mTOR signaling to promote vascular rejuvenation, improve NO-mediated endothelium-dependent vasodilation and attenuate vascular oxidative stress in aged mice (95). Plasma HDL content decreases with age. Ben J Wu et al. demonstrated that young plasma inhibits endothelial inflammation and decelerates vascular endothelial cell senescence through HDL-promoted 24-dehydrocholesterol reductase (DHCR24) expression, which in turn mediates PI3K/AKT/heme oxygenase-1 (HO-1) signaling pathway (96). In addition, Xiaorui Chen et al. demonstrated that young EVs not only improves cognitive function, counteracts mitochondrial defects, and enhances *in vitro* respiratory capacity by stimulating PGC-1α expression and enhancing mitochondrial energy metabolism, but also rejuvenates senescent tissues and inhibits cellular senescence, which is an indication of the ability of young EVs to ameliorate vascular endothelial cell aging (48).

### 5.5 Inhibit cardiac inflammation and oxidative stress

Blood is a fluid tissue composed of plasma and blood cells, and plasma is the liquid component of blood, accounting for about 55% of the blood volume. Shiyao Wei et al. found that young plasma by decreasing the expression of pro-inflammatory chemokines in angiotensin II-stimulated pericytes which are located between the endothelial cells of capillaries and the basement membranes, and which have physiological functions such as regulating vascular function, participating in angiogenesis and immunomodulation), the pro-inflammatory and pro-fibrotic effects of angiotensin II on pericytes can be ameliorated (97), thus contributing to attenuating the effects of inflammation on the development and progression of HF. Kai Chen et al. found that exogenously secreted Klotho promotes the expression of GR through Nrf2 activation, and verified that cardiac-specific expression of GR can diminish oxidative stress and apoptosis in the hearts of aged mice, with a protective effect against age-related cardiac injury (71). Shaina Ailawadi et al. found that multiple sources of stem cells, stress preconditioned cells, and genetically modified cells can generate cardioprotective exosomes to mitigate inflammation and oxidative stress in the heart (98, 103).

## 6 Discussion

HF remains one of the major challenges in modern medicine. In recent years, young blood has emerged as a promising research frontier with substantial potential for treating age-related diseases such as HF. Endi Xia et al. demonstrated that intravenous injection of young blood could alleviate hippocampus-dependent learning and memory deficits, and restore synapse formation and synaptic plasticity in mice with Alzheimer’s disease, which proved that young serum had a therapeutic effect on it (104). Anding Liu et al. demonstrated that infusing of young blood into aged mice alleviates aging-induced liver injury, liver fibrosis and reduces hepatocyte senescence (105). Shi-Yao Wei et al. also demonstrated that systemic administration of young plasma reduces the progression of renal fibrosis and the transition from acute injury to chronic renal disease et al. (97). Alkahest, a derivative of young blood, which was invested in and developed by Grifols, the world’s largest plasma manufacturer, has been used in preclinical studies of Alzheimer’s disease (106). Although Alkahest and similar young blood derivatives are still in the development and experimental stages, they still show great therapeutic promise and potential for young blood.

The great therapeutic potential and distinct advantages of young blood are reflected in the following aspects: (i) The multiple rejuvenating circulating factors in young blood offer the possibility of personalized treatment for HF. HF patients exhibit significant etiological heterogeneity and pathophysiological diversity, which traditional treatment protocols struggle to address uniformly. In contrast, young blood’s multi-component, multi-target synergistic effects enable tailored intervention across distinct pathological dimensions of HF. By leveraging biomarker-guided screening of active components and dose optimization, young blood therapies can be further integrated with other therapeutic modalities to establish precision-driven, personalized strategies aligned with each patient’s unique pathological profile. (ii) Circulating factors and mediators in young blood represent endogenous substances naturally produced by the body, functioning within normal physiological environments to ensure excellent biocompatibility and minimal immunogenicity. In contrast to synthetic therapeutic drugs or biologics, young blood-derived components carry a relatively lower risk of triggering immune rejection or other adverse reactions, enhancing patient tolerability. This inherent safety profile renders them an ideal candidate for long-term, safe management of HF, particularly for elderly patients or vulnerable populations with multiple comorbidities—while potentially mitigating treatment-related risks of dose dependency. (iii) The exploration of young blood transcends the limitations of traditional aging-related research and therapies, which typically focus on single symptoms or isolated pathological aspects of diseases. Unlike conventional approaches, young blood’s multi-component nature enables holistic, multi-target and multi-pathway improvements in cardiac and other tissue or organ functions. By modulating the organism’s overall aging trajectory, this strategy not only ameliorates age-related conditions like HF but also offers novel paradigms for preventing and treating aging-related liver and kidney diseases. This shift redirects research focus from merely treating diseases to proactively intervening in the organism’s systemic aging process, with the potential to redefine the research, prevention, and treatment paradigms for all aging-related disorders.

Although young blood shows great potential in improving the function of the failing heart, there are still some limitations in the current study. (i) The understanding of the role of young blood in cardiac regulation remains limited, primarily constrained by the interactions between various circulating factors and their specific mechanisms of action in regulating cardiac function. First, these circulating factors may regulate cardiac function through complex synergistic or antagonistic interactions among them, but the relative contribution of each factor and its dose-dependent effects still lack corresponding studies. Second, the current identification of key effectors is mainly based on a single factor research paradigm, and the relationship with young blood as a whole remains to be investigated. Third, directly infusing young blood for therapeutic purposes may carry risks such as immune rejection, allergic reactions and transmission of infections. Fourth, young blood’s components may instead exacerbate the progression of the disease, such as high levels of GDF11 cause severe cachexia, death, cardiac and skeletal muscle wasting (107), TNF-α and IL-6 promote cardiac remodeling and inflammation generation (108). Finally, current research on the subtype-specific effects of young blood and its circulating factors in HF is still in its preliminary stage. As noted, ARNI is recommended for HFrEF, while GDF11 and EVs synergistically improve ventricular systolic function via anti-myocardial hypertrophy and inhibiting myocardial fibrosis, respectively. SGLT2i is recommended for HFpEF, with Klotho reducing myocardial cell damage and NO alleviating endothelial vascular resistance elevation. (ii) The donor screening of young blood, the identification and isolation operation of active ingredients and regulatory issues regarding blood-derived therapies have not yet established unified standards. This makes it difficult to obtain circulating factors that meet the requirements of experimental research and clinical treatment, which affects the accuracy of the research results and the therapeutic effects in the clinic. Since most of the relevant studies focus on animal models, which have significant physiological differences from humans, leading to a scarcity of clinical data and insufficient evidence for the safety of young blood in clinical applications. (iii) The technology of artificially synthesizing active ingredients related to young blood has not yet achieved a breakthrough, and it is difficult to accurately construct their molecular structures with the existing biosynthesis technology, making it is impossible to satisfy the needs of a large number of patients relying on the blood of the donor alone. This may even give rise to ethical and social issues such as violations of donors’ health and damage to healthcare equity.

There is no dispute regarding the anti-aging effects of young blood, especially its therapeutic potential in HF. Future research should also focus on the following aspects: (i) In-depth mechanistic studies can help to clarify the relationship between the key effectors and young blood, and between young blood and HF subtypes. With the help of proteomics, metabolomics and other technologies to clarify the intrinsic relationship between the key effectors and young blood, analyze the specific effect mechanism of the key effectors on the heart and other aging tissues and organs. As well as explore the combined effects of young blood and existing therapeutic drugs and establish optimal dosage thresholds to avoid potential risks of young blood transfusion therapy. (ii) Unify the screening criteria for young blood donors, develop standardized procedures for the collection, storage and use of young blood, and strengthen the approval standards and supervision of blood therapies, while more in-depth techniques such as high-resolution proteomics and single-cell sequencing should be used for the identification and separation of active ingredients to accurately identify and extract the active ingredients in young blood. Furthermore, human clinical trials should be initiated through approaches such as organoid models and optimized administration dosages and methods to accumulate clinical data for therapeutic applications, validate the safety, efficacy and patient-specific responsiveness of young blood-based interventions, so as to accelerate the translation from existing animal experiments model to clinical practice. (iii) Strengthen the cooperation among multiple disciplines, such as biochemistry, cell biology, computer science, etc., and increase the funding and research investment on the artificial synthetic technology of active ingredients in young blood, focusing on the structural analysis and biosynthetic pathway of active ingredients, so as to make every effort to avoid the ethical and social problems caused by the direct infusion of young blood.

## 7 Conclusion

As a momentous breakthrough within the realm of anti-aging medicine, young blood therapy demonstrates unique advantages in enhancing cardiac function through the coordinated actions of multiple targets and pathways. The therapy transcends the limitations of traditional single-target therapy and offers an entirely novel intervention strategy and treatment paradigm for the management of HF and age-related maladies. However, there remain translational barriers from basic research to clinical application, as the complex interactions among various components of young blood, the lack of standardized systems, and ethical issues in clinical translation are urgently to be addressed. Future research should focus on current gaps. It is necessary to deeply analyze the dynamic interaction network of key circulating factors, clarify their synergistic or antagonistic effects and molecular regulatory pathways in different pathological microenvironments. Additionally, it is need to systematically evaluate the applicability of young blood therapy in special populations and improve risk early-warning mechanisms through dynamic monitoring of immunogenicity and long-term physiological impacts during treatment, thus gradually overcoming the core bottlenecks in translating basic research to clinical practice.
